# Automated MSCT Analysis for Planning Left Atrial Appendage Occlusion Using Artificial Intelligence

**DOI:** 10.1155/2022/5797431

**Published:** 2022-04-27

**Authors:** Kilian Michiels, Eva Heffinck, Patricio Astudillo, Ivan Wong, Peter Mortier, Alessandra Maria Bavo

**Affiliations:** ^1^FEops NV, Gent, Belgium; ^2^The Heart Centre, Rigs Hospitalet, Copenhagen University Hospital, Copenhagen, Denmark

## Abstract

**Background:**

The number of multislice computed tomography (MSCT) analyses performed for planning structural heart interventions is rapidly increasing. Further automation is required to save time, increase standardization, and reduce the learning curve.

**Objective:**

The purpose of this study was to investigate the feasibility of a fully automated artificial intelligence (AI)-based MSCT analysis for planning structural heart interventions, focusing on left atrial appendage occlusion (LAAO) as the selected use case.

**Methods:**

Different deep learning models were trained, validated, and tested using a cohort of 583 patients for which manually annotated data were available. These models were used independently or in combination to detect the anatomical ostium, the landing zone, the mitral valve annulus, and the fossa ovalis and to segment the left atrium (LA) and left atrial appendage (LAA). The accuracy of the models was evaluated through comparison with the manually annotated data.

**Results:**

The automated analysis was performed on 25 randomly selected patients of the test cohort. The results were compared to the manually identified landmarks. The predicted segmentation of the LA(A) was similar to the manual segmentation (dice score of 0.94 ± 0.02). The difference between the automatically predicted and manually measured perimeter-based diameter was −0.8 ± 1.3 mm (anatomical ostium), −1.0 ± 1.5 mm (Amulet landing zone), and −0.1 ± 1.3 mm (Watchman FLX landing zone), which is similar to the operator variability on these measurements. Finally, the detected mitral valve annulus and fossa ovalis were close to the manual detection of these landmarks, as shown by the Hausdorff distance (3.9 ± 1.2 mm and 4.8 ± 1.8 mm, respectively). The average runtime of the complete workflow, including data pre- and postprocessing, was 57.5 ± 34.5 seconds.

**Conclusions:**

A fast and accurate AI-based workflow is proposed to automatically analyze MSCT images for planning LAAO. The approach, which can be easily extended toward other structural heart interventions, may help to handle the rapidly increasing volumes of patients.

## 1. Introduction 

During the last decade, there has been an exponential growth in the number of structural heart interventions, largely driven by the widespread adoption of transcatheter aortic valve replacement (TAVR) [1]. A continued growth can be expected in the coming years due to a further expansion of TAVR in combination to significantly increase volumes for several other interventions, such as left atrial appendage occlusion (LAAO) and transcatheter mitral valve repair and replacement (TMVR).

Medical imaging is of utmost importance for all these structural heart interventions, from preprocedural planning to intraprocedural guidance and postprocedural follow-up. A wide variety of imaging modalities can be used during these different stages. Notably, many centers rely on multislice computed tomography (MSCT) for preprocedural planning [2, 3]. Driven by the enormous growth in structural heart interventions, there has been a steep increase in the number of MSCT analyses that need to be performed. Given that the currently available software solutions only provide semi-automated workflows, further automation is required. This may not only help to save a considerable amount of time but can also result in more standardization and a shorter learning curve for a starting operator.

An MSCT analysis for planning structural heart interventions—whether this is TAVR, LAAO, or any other procedure—typically requires identifying certain anatomical landmarks and deriving measurements from these landmarks, in order to assess the procedural risks and to guide device selection and sizing. In addition, a segmentation or 3D volume reconstruction of certain anatomical structures is sometimes performed to better understand the patient's anatomy. With the recent advances in artificial intelligence (AI), it has become possible to automate all these tasks (landmark identification, measurements, and 3D reconstruction) [4, 5].

The aim of this study was to investigate the feasibility of a fully automated AI-based MSCT analysis for planning structural heart interventions, focusing on LAAO as the selected use case.

## 2. Methods

This retrospective study was performed using MSCT images acquired for the preoperative planning of the LAAO. The full cohort contains 583 distinct MSCT datasets collected from 41 different medical centers. The patient cohort is characterized by a mean age of 76.5 ± 7.9 years, and 44.9% of male and 24.7% of female patients (gender unknown in 30.4% of the patients).

### 2.1. MSCT Analysis for LAAO in Clinical Practice

**Figure 1:**
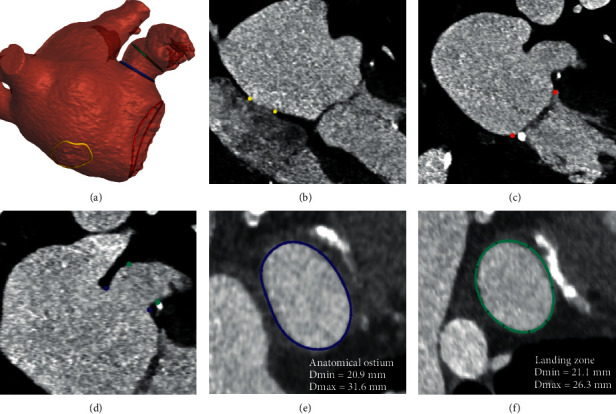
Anatomical structures and landmarks identified by the model. (a) 3D model reconstructed from the segmentation of the left atrium and left atrial appendage, where the landmarks of the anatomical ostium (blue), landing zone (green), fossa ovalis (yellow), and mitral annulus (red) are reported. (b) Fossa ovalis region indicated on the DICOM (yellow). (c) Mitral annulus indicated on the DICOM (red). (d) Anatomical ostium and landing zone indicated on the DICOM (blue and green, respectively). (e) Anatomical ostium plane. (f) Landing zone plane (Amulet device).

A typical MSCT analysis for the preprocedural planning of LAAO involves several aspects [3, 6]. The size of the left atrial appendage (LAA) is assessed by identifying the 3D planes defining the entrance of the LAA (i.e., anatomical ostium) and a device-specific landing zone, and by performing measurements in these planes. The depth of the appendage is also measured, to understand whether the LAA is deep enough to host the selected device. To plan the transseptal puncture site, the fossa ovalis is identified as a 3D curve on the interatrial septum. Locating the mitral valve annulus can also be useful to assess whether there could be any potential interaction between the implanted occluder and the mitral valve. Finally, a 3D model reconstruction of the left atrium (LA) and the LAA is often generated through image segmentation techniques to better understand the patient's anatomy. The described anatomical landmarks and measurements are depicted together with the 3D model of the LA(A) in Figure 1.

### 2.2. Manual MSCT Analysis Available as Ground Truth

Manually annotated or “ground truth” data have been produced by trained professionals for all the abovementioned landmarks and the 3D segmentation of the LA(A), using the Materialise Mimics Innovation Suite 21 (Materialise, Leuven, Belgium), according to the indications provided in the instruction for use of the devices and in the most relevant literature of the field [3]. Not all annotations are available for all patients as some were added at a later stage of the study.

For 25 patients, three trained professionals identified the ostium and landing zone planes independently and performed the related measurements. This provides interoperator variability data that allow to correctly interpret the accuracy of the deep learning models.

### 2.3. Automated MSCT Analysis

**Figure 2:**
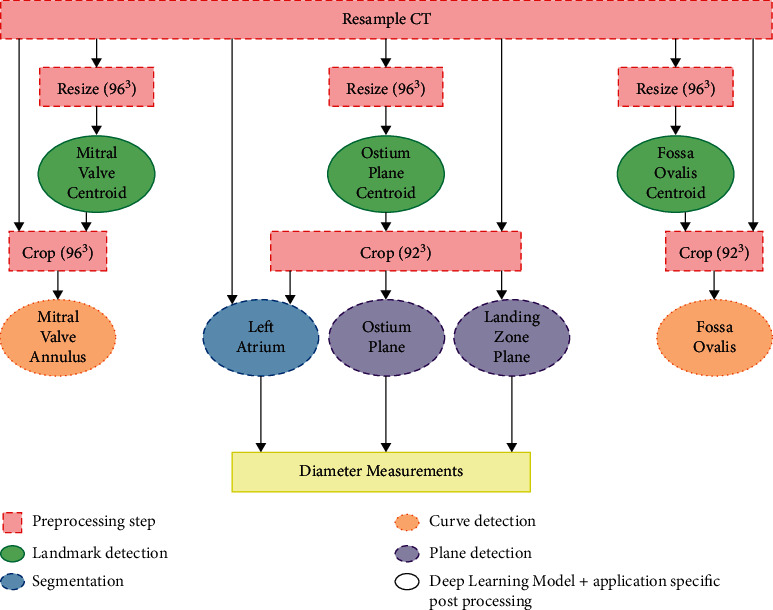
Overview of the steps included in the complete workflow.

Four distinct application types based on deep learning are used independently or in combination to provide the required output for the anatomical analysis: segmentation, point detection, curve detection, and plane detection. The complete data flow, starting from the resampled MSCT data, is shown in Figure 2. The deep learning models used here are based on the NiftyNet implementation [6] (variations of DenseVNet [5]), where the prior and the initial average pooling layer can be omitted. Other strategies were investigated, but none gave comparable results in terms of accuracy.

For each application, the amount of data used for the training, validation, and testing of the deep learning models was 80%, 10%, and 10%, respectively. Data were randomly distributed over these three different groups.

The train and validate set are used during the training and hyperparameter optimization of the deep learning models, while the test set is an “unseen” dataset used to assess model accuracy. For the purpose of this manuscript, a fixed group of 25 randomly selected patients was used in the test cohort for all applications. The average age in this test set is 77.35 ± 8.22 years. The gender distribution is 52% of male patients, 16% of female patients, and 32% of unknown patients. For the same 25 patients, the manual annotations obtained from different operators were used in the interoperator variability study to assess the accuracy of the automated ostium and landing zone plane detection and the related anatomical measurements. This condition does not alter the data distribution across the groups, but it ensures a meaningful comparison of the results.

**Figure 3:**
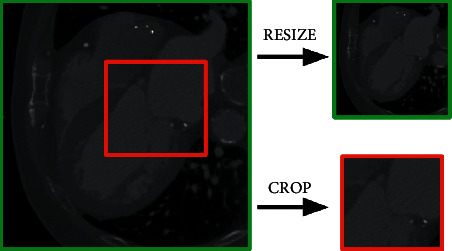
CT data can be resized (top right) or cropped (bottom right) depending on the application at hand. Resizing keeps the entire data but in a smaller format. Cropping takes out a region of interest without any resizing.

In order to automatically perform the above-described anatomical analysis for LAAO, a preprocessing step of the MSCT data is required. Initially, one cardiac phase has to be selected. This step is performed manually, and it is not included in the “automatic analysis workflow” described here. As part of the automatic process, firstly the MSCT volume needs to be resampled to an isotropic resolution and voxel size (different values depending on the specific application). Once the MSCT volumes are isotropic, they are resized or cropped to an application-specific input shape. The difference between resizing and cropping is illustrated in Figure 3.

The following sections provide more details regarding the four different types of applications.

#### 2.3.1. Segmentation

**Figure 4:**
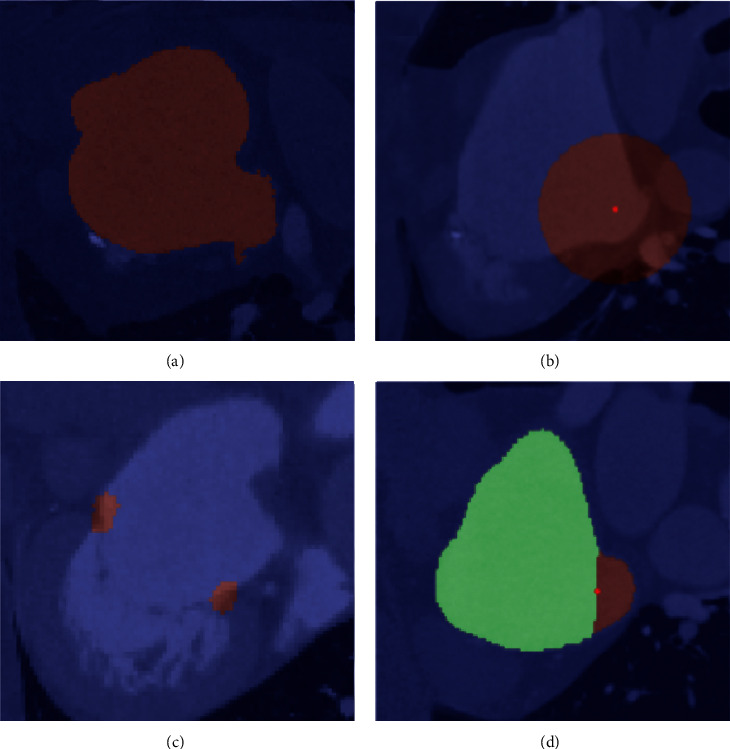
Different ground truth masks created for the AI model trainings, overlaid on top of the DICOM images. (a) Segmentation of the left atrium and left atrial appendage. (b) Point detection, where the dark red region (label) represents the spherical region around the point, and the bright red dot is the centroid of the mask identified as output. (c) 3D curve detection. (d) Plane detection, where the different labels are identified with different colors.

Segmentation is the task of assigning a specific label to each part of the input. In this case, the input is a 3D volume and the segmentation output is a 3D volume of the same shape, with a label identifier assigned to each voxel inside the volume. The manually obtained segmentation masks describe which voxels are part of the LA(A), and these data are used to train a deep learning model. An example of the LA(A) mask is shown in Figure 4(a). When applying the trained deep learning model, a probability mask is returned, describing the probability that a certain voxel belongs to the LA(A) label. Postprocessing of the model output is required to binarize the obtained probability mask. Given a threshold, all probabilities in the mask below this threshold are set to label zero, while all values equal to or higher than the threshold are set to label one. The resulting segmentation mask is the volume described by all the voxels with label one. To obtain a higher precision mask, the deep learning mask is combined with masks obtained through image analysis techniques (such as water shedding).

#### 2.3.2. Point Detection

In order to detect a 3D point within the MSCT volume, the location of the manually identified point is used to generate a segmentation mask by assigning a predefined label to a spherical region around the point (highlighted in dark red in Figure 4(b)). Deep learning models are then trained to return a probability mask of that same region. Postprocessing is similar as compared to the segmentation application, with the additional step of taking the centroid of all the similar labels to obtain a 3D point. This 3D point detection is used to identify specific regions of interest in the MSCT data for further processing as shown in Figure 2. For example, the centroid of the mitral valve is detected in order to crop the MSCT data around the mitral valve, which is then used to identify the mitral valve annulus. This application type is based on the work of Astudillo et al. [8].

#### 2.3.3. Curve Detection

The manually identified curves (fossa ovalis or mitral annulus) are used to generate a segmentation mask by sweeping a sphere along the curve with an application-dependent radius. This results in a torus-shaped segmentation mask (Figure 4(c)). The probability mask returned by a trained deep learning model is transformed into a 3D curve using graph-based techniques, as described in the work of Astudillo et al. [9].

#### 2.3.4. Plane Detection

Plane detection is fundamental to derive the diameter measurements used by physicians to understand the size of the LAA. The manually identified planes (such as the anatomical ostium and landing zone) are used to split the manually obtained LA(A) segmentation mask into two regions, as shown in Figure 4(d). Using this input, deep learning models are trained to assign voxels within the LA(A) to one of these two labels. Subsequently, the connecting boundary between the voxels annotated by these labels can be extracted using imaging processing techniques and used to fit a plane.

### 2.4. Derived Measurements

Using the items described above, additional output required for the preoperative planning can be extracted. For each of the detected planes (ostium and landing zones), a closed curve describing the boundary of the appendage in the predicted planes is derived using the LA(A) segmentation and four diameters are calculated (area-based, perimeter-based, minimum, and maximum diameters).

The LAA depth (for Amulet devices) can also be derived, calculated as the distance between the centroid of the anatomical ostium plane and its projection to the LAA surface, at the roof of the LAA. With a similar procedure, the LAA depth (for Watchman FLX devices) can be derived, by calculating the distance between the landing zone centroid and the LAA tip.

### 2.5. Evaluation Metrics

Depending on the application, the prediction is evaluated using different metrics. Segmentations are evaluated by the Sørensen–Dice coefficient [10, 11], while for point detections, the Euclidean distance between the predicted and ground truth curves is used.

The curve detection models are assessed with the Euclidean distance between the centroids of the predicted and ground truth curves. This metric provides information about the accuracy of the location of the detected curve. In addition, the Hausdorff distance [12] and the difference in diameter of the predicted and ground truth curves are calculated to assess the accuracy of the shape of the curve.

The detected planes are evaluated using the angle between the predicted and ground truth planes. In addition, the Euclidean distance between the centroid of the closed curve describing the boundary of the appendage in the predicted and ground truth planes is calculated to assess the location error.

### 2.6. Quality Control

For the purpose of this manuscript, the results reported in the following sections do not include any quality check step or manual modifications, to ensure that the accuracy of the models is calculated without any subjective corrective action. The processing time reported refers to the automatic tasks only, even though manual steps (e.g., phase selection) are still required in the preprocessing phase.

## 3. Results

The automated analysis was completed for the patients included in the test cohorts (*n* = 25). The average runtime of the complete workflow, including data pre- and postprocessing, was 57.5 ± 34.5 seconds when executed on a GPU server with 4 GPUs (2x Nvidia GeForce RTX 2080 ti, 1x Nvidia GeForce RTX 2070 SUPER, and 1x GeForce GTX TITAN X) and 64 GB RAM, using TorchServe [13]. The time spent by qualified professionals to perform the same tasks manually was approximately 10–15 minutes per patient.

The accuracy of the different applications is provided in the following paragraphs. For each patient, the comparison between the automatic and the manual analyses has been performed on images of the same cardiac phase.

### 3.1. LA(A) Segmentation

The mask resulting from the deep learning models and the image analysis techniques is evaluated for the 25 patients on whom the interoperator variability study was performed. The mean Dice score is 0.94 ± 0.02.

### 3.2. Plane Detections and Related Measurements

**Figure 5:**
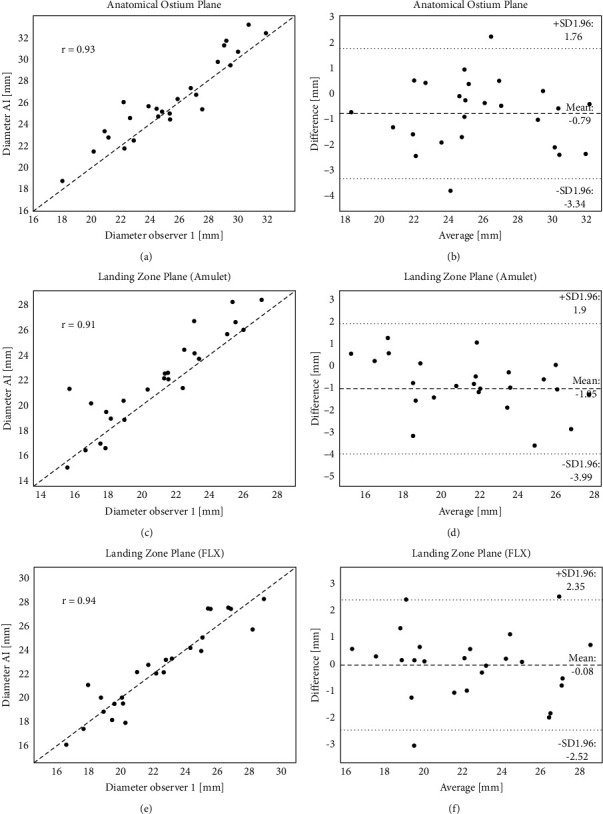
Overview of the results obtained for the comparison between the AI models and one of the manually identified measurements. All graphs report the results obtained for the perimeter-based diameter of the indicated cross-section (a-b: anatomical ostium, c-d: landing zone Amulet, e-f: landing zone Watchman FLX). Graphs (a-c-e) scatter plot with R Pearson coefficient. Graphs (b-d-f) Bland–Altman analysis with mean value and limits of agreement.

**Figure 6:**
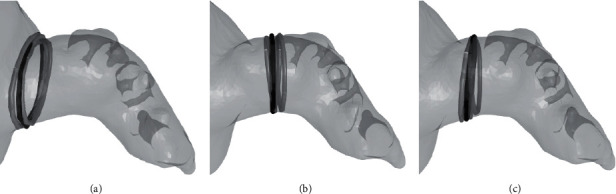
Example of the ostium and landing zone curves in the detected planes for 1 patient. The black curve shows the predicted curve, while the curves from the three different observers are shown in gray. (a) Anatomical ostium plane. (b) Landing zone plane (Amulet). (c) Landing zone plane (Watchman FLX).

The prediction of the anatomical ostium and landing zone planes, as well as the resulting anatomical measurements, is evaluated using the interoperator variability data that were conducted on 25 patients.  Table 1 provides an overview of all the results using the data from observer 1 as the comparator. It can be observed that the differences between the model predictions and observer 1 are very similar to the differences between the different observers, both in terms of the derived measurements as well as for the location and orientation of the detected planes. Scatter and Bland–Altman plots are provided in Figure 5 for the perimeter-based diameter at the ostium and the different landing zone planes.  Figure 6 shows the manually identified and predicted curves for one randomly selected patient.

### 3.3. Mitral Valve Annulus

**Figure 7:**
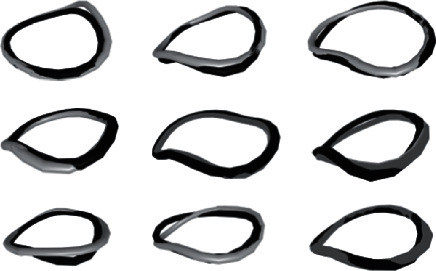
Mitral valve annulus curves for nine randomly selected patients of the test dataset. The manually detected and the predicted curve are displayed in gray and black, respectively.

The mean diameter difference of the detected mitral valve annulus is 0.1 ± 0.9 mm for the test set, while the mean Hausdorff distance is 3.9 ± 1.2 mm. This means that the shape of the predicted mitral valve annulus is accurately predicted. The location error is represented by the mean distance error between the ground truth and the centroids of the predicted curve. This error is 1.2 ± 0.8 mm and confirms the location accuracy of the predicted curve.  Figure 7 shows a qualitative comparison of the predicted and ground truth mitral annulus curves for nine randomly selected patients included in the test set.

### 3.4. Fossa Ovalis

For the test set, the fossa ovalis mean diameter difference is −2.7 ± 4.2 mm, with a Hausdorff distance of 6.7 ± 5.1 mm. The Euclidean distance error on the centroid of the curve is 4.1 ± 5.0 mm. Of note, the region of the fossa ovalis is clearly visible only if there is sufficient contrast filling in the right atrium. The MSCT acquisition protocols vary from center to center, and not for all patients the contrast sufficiently reaches the right atrium for the identification of a proper fossa ovalis. This explains why for the fossa ovalis the performance of the model is lower than for the mitral annulus. When excluding from the analysis the 4 DICOM datasets with poor contrast filling in the right heart, the mean diameter difference is reduced to −2.1 ± 3.0 mm, with a Hausdorff distance of 4.8 ± 1.4 mm. The Euclidean distance error on the centroid of the curve is 2.3 ± 1.0 mm.

**Figure 8:**
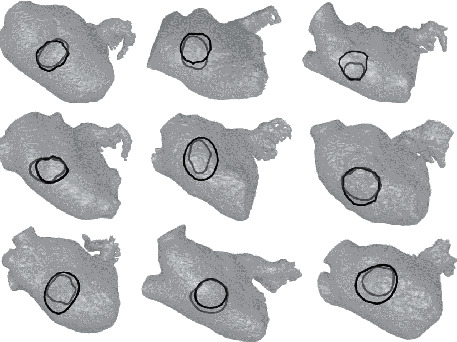
Fossa ovalis curves for nine randomly selected patients from the test dataset. The manually detected and the predicted curve are displayed in gray and black, respectively.

In Figure 8, a qualitative comparison of the prediction and the ground truth is given for nine patients randomly selected from the test set.

## 4. Discussion

### 4.1. Preoperative LAAO Anatomical Analysis Tool

Several AI-powered models have been reported in the literature [14, 15], and tools and platforms are described offering semi-automated analysis, based on 3D echocardiography [16] and MSCT images [17]. Commercially available software exists, allowing for a predefined workflow for the preoperative planning of LAAO procedures, where the physician still needs to interact with the tool and provide manual input to the software.

In this work, we presented a framework consisting of several AI-based applications, to allow for the automatic anatomical analysis needed for the preoperative planning of the LAAO. After the preprocessing phase to ensure image selection and standardization, no interaction or input is required to generate the results. The proposed method is based on MSCT scans, which provide high spatial resolution. The availability of larger portions of the heart compared to 3D echocardiography allows the inclusion of relevant structures such as the fossa ovalis contour, for transseptal puncture planning. The proposed method is independent from the origin of the data, MSCT machinery manufacturers, and MSCT acquisition protocol, as the model has been developed and tested on a large database spanning a wide range of parameters for the abovementioned characteristics.

The presented framework is fast (1 minute vs. 10–15 minutes of manual work), accurate, and is built on a large database (>500 MSCT scans), providing a solid base for the AI-based models. This framework can easily be extended to other structural heart disease interventions. The availability of such an analysis for physicians ensures a fast and accurate anatomical analysis, which is crucial for a successful and efficient LAAO procedure.

Clinically, as the LAAO procedure is still not as widespread as TAVR, the learning curve of preprocedural planning in low-volume centers can be long and difficult. The availability of an automatic tool for the preoperative anatomical analysis may not only result in more standardization across different operators but may also shorten the learning curve during initiation of the programs.

### 4.2. Quality Control and User Interaction

As stated before, all the results presented here are calculated in a fully automated manner, to prove the accuracy of the models. When the applications described are translated into clinical practice tools, the interaction with the user or the physician remains fundamental. As the preoperative planning of a procedure relies on the extensive experience of the operator, it is the authors' vision that the physician should always be able to interact with the provided results, and to modify them if needed. For example, a way to deliver the AI results would be the inclusion of the described models into a user-friendly interface, where the operator can inspect, review, and modify the preoperative landmarks and measurements if needed.

Furthermore, to ensure the applicability of the developed methodology regardless of infrastructure limitations, such a model could be integrated into a cloud-based service/platform, which is easily accessible and removes several constraints on hardware availability and maintenance.

### 4.3. Extension to Other Fields of Application

The work presented for LAAO preprocedural planning serves as a use case to demonstrate the availability, accuracy, and speed of the developed AI-based applications. Additional features to the workflow can be easily integrated, to expand the preoperative planning even further. Relevant additions are the LAA centerline detection, to understand the tortuosity of anatomies and the positioning of the delivery system; to investigate the trajectory between the transseptal puncture location and the access to the LAA, and computational simulations [18]; and to calculate the physical interaction between the virtually deployed device and the anatomical structures.

Similar algorithms can be used for other interventions, where preoperative planning of transcatheter procedures based on MSCT images is mandatory. For TAVR, this may be very useful considering the large number of MSCT analyses that need to be performed in high-volume centers [4]. It also has the potential to significantly speed up the planning of procedures such as TMVR, where multiple analyses at different phases of the cardiac cycle are required, resulting in a relatively time-consuming process [7, 9].

### 4.4. Current Limitations

The current study logically has some limitations. The interoperator variability study conducted as a comparator included only a limited cohort of patients (*n* = 25). For a stronger comparison and dedicated statistical subanalyses to detect potential patterns in the automated landmark detection, a larger cohort of patients should be analyzed by qualified operators.

From a clinical point of view, the models have been presented and validated for the LAAO use case. The extension to other structural heart interventions might require the implementation of additional models, to deliver all the relevant landmarks and parameters necessary for the planning of the corresponding procedures.

## 5. Conclusion

This manuscript presents a fast and accurate AI-based workflow, to automatically analyze MSCT images for preprocedural planning of LAAO interventions. The approach, which can be easily extended to other structural heart interventions, may help to handle the rapidly increasing volumes of patients, to speed up the manual process of anatomical analysis, and to facilitate the preoperative planning for transcatheter procedures.

## Figures and Tables

**Table 1:** Overview of the differences between the manual analysis from observer 1 (obs1), the model predictions, and the manual analyses of observer 2 (obs2) and 3 (obs3). The differences are reported as mean ± standard deviation.

	Model vs. obs1	Obs2 vs. obs1	Obs3 vs. obs1
Anatomical ostium plane			
Area-based diameter (mm)	−0.8 ± 1.3	−0.8 ± 1.2	−0.4 ± 1.1
Perimeter-based diameter (mm)	−0.8 ± 1.3	−0.8 ± 1.3	−0.4 ± 1.2
Maximal diameter (mm)	−0.9 ± 2.0	−0.9 ± 1.6	−0.6 ± 1.6
Minimal diameter (mm)	−0.6 ± 1.1	−0.6 ± 1.1	−0.1 ± 0.8
Centroid (mm)	1.9 ± 1.0	1.9 ± 0.9	1.7 ± 0.7
Angle [°]	6.5 ± 2.9	6.0 ± 3.0	6.5 ± 3.4

Landing zone plane (amulet)			
Area-based diameter (mm)	−0.9 ± 1.5	−0.2 ± 0.6	0.3 ± 1.0
Perimeter-based diameter (mm)	−1.0 ± 1.5	−0.2 ± 0.6	0.3 ± 1.0
Maximal diameter (mm)	−1.2 ± 2.0	−0.4 ± 1.1	0.2 ± 1.3
Minimal diameter (mm)	−0.6 ± 1.7	0.0 ± 0.9	0.6 ± 0.9
Centroid (mm)	1.8 ± 1.1	1.7 ± 0.9	1.5 ± 0.8
Angle [°]	8.3 ± 5.1	6.6 ± 3.7	8.9 ± 3.6

Landing zone plane (Watchman FLX)			
Area-based diameter (mm)	−0.1 ± 1.2	0.2 ± 1.0	0.7 ± 0.9
Perimeter-based diameter (mm)	−0.1 ± 1.3	0.1 ± 1.1	0.8 ± 1.0
Maximal diameter (mm)	0.1 ± 1.7	0.2 ± 1.9	0.9 ± 1.5
Minimal diameter (mm)	−0.2 ± 1.4	0.0 ± 0.9	0.6 ± 1.0
Centroid (mm)	1.8 ± 1.5	2.0 ± 1.3	2.0 ± 1.0
Angle [°]	7.8 ± 5.1	7.7 ± 4.7	8.4 ± 4.9

## Data Availability

The statistical data used to support the findings of this study are available from the corresponding author upon request.
